# The Influence of a School Social Network Intervention on Adolescent's Health Behaviors: A Gender-Specific Agent-Based Model

**DOI:** 10.3389/fpubh.2022.861743

**Published:** 2022-04-04

**Authors:** Shu Zhang, Tianyi Xiao, Jie He

**Affiliations:** ^1^School of Architecture, Tianjin University, Tianjin, China; ^2^School of Architecture, Harbin Institute of Technology (Shenzhen), Shenzhen, China

**Keywords:** gender differences, agent-based model (ABM), obesity, peer influence, small-world networks, centrality measurement

## Abstract

**Introduction:**

Adolescence is a crucial stage for health behavior development, which is associated with health in adulthood. School closures caused by the coronavirus disease 2019 (COVID-19) pandemic have exposed adolescents to an increased risk of obesity due to a lack of physical activity. Although social network interventions provide an effective approach for promoting health-related behavior, current practices neglect gender differences in adolescent behavioral patterns and emotional preferences. The aim of this study was to examine the effectiveness of centrality-based methods integrated with of gender contexts in a social network intervention to improve adolescent's health behavior.

**Methods:**

We developed an agent-based model (ABM) that supports the small-world characteristics of adolescent social networks. Health-related data for junior middle school students (*n* = 234, 48% girls) were collected in November 2018, 2019 and 2020 in Tianjin, China. We simulated multiple network-based interventions with different criteria for influential agents (i.e., betweenness centrality, closeness centrality, eigenvector centrality, and PageRank) and a random condition. The rules for generating peer influence and accelerating behavioral changes were based on the diffusion of innovations theory, with gender specifications.

**Results:**

After the school closures, there was a significant increase in the prevalence of overweight and obesity among adolescents, with a greater increase in girls than in boys (+8.85% vs. +1.65%, *p* < 0.001). Simulations showed that centrality-based network interventions were more effective than the random condition (average 6.17% per tick vs. 5.22% per tick, *p* < 0.05), with a higher efficiency in girls than boys (average 3.68% vs. 2.99% per tick, *p* < 0.05). PageRank outperformed other centrality conditions at the population level (6.37% per tick, p < 0.05). In girls, betweenness centrality was the best method (3.85% per tick, *p* < 0.05), while in boys, PageRank still had the greatest efficiency (3.21% per tick, *p* < 0.05).

**Conclusions:**

We found evidence for gender differences in the negative impact of COVID-19-related school closures and the potential for centrality-based social network interventions to affect adolescent health behavior. Therefore, we emphasize the importance of gender-specific targeting strategies to further promote health-related school programs in the post-pandemic era.

## Introduction

Adolescent's patterns of health behavior, especially physical activity (PA), are associated with academic achievement (1) and lifelong health in adulthood, including obesity and related diseases (2). The World Health Organization (WHO) recommends that schoolchildren aged 5–17 years accumulate at least 60 min of moderate-to-vigorous physical activity (MVPA) daily (3). However, nearly 80% of adolescents aged 13–15 worldwide do not meet these guidelines (4), resulting in increasing rates of overweight and obesity in adolescents (5, 6). Recently, temporary school closures due to the coronavirus disease 2019 (COVID-19) pandemic have exacerbated physical inactivity and obesity problems (7) and have raised broad social concern about the promotion of positive adolescent health behavior.

Schools offer an appropriate environment for adolescents to shape and sustain health-related behaviors. These behaviors may arise and be reinforced through observing and imitating peers (8). Peer influence, which emphasizes the friendship ties in social relationships (9), plays a crucial role in increasing PA. Evidence suggests that adolescents behave more actively when together with their peers (10), and they influence the PA level of their friends (11). As peer influence is often recognized as a social network phenomenon (12), studies have used social network interventions for various health behavior issues, such as smoking prevention (13), PA promotion (14), and water consumption (15). The results of these studies indicate the efficiency of social network interventions in accelerating individual behavioral change and improving collective health performance by a shared physical environment.

Social network intervention is based on the diffusion of innovations theory (16). Using social network data, interventions are developed with multiple tactical alternatives, including individuals, segmentation, induction, and alteration. The selection of these alternatives is based on data availability, the perceived characteristics of the behavior, the existing prevalence, and the social context (17). Studies of social networks have highlighted the value of network structure, indicating that a highly centralized network may benefit from using leadership strategies (18, 19) to inform leader selection criteria or by targeting the most influential individual, which can be provisionally determined by different concepts of centrality (i.e., in-degree centrality, closeness centrality, and betweenness centrality) (20). Van Woudenberg et al. (21) found that a network-based intervention that used closeness centrality to select influential individuals outperformed the betweenness centrality condition in promoting PA in a school setting. However, there is limited guidance on gender contexts in health behavior strategies, which may have contributed to the unpredictable process and analysis results of previous interventions (22).

There is increasing evidence for gender differences in PA participation and changes in weight among adolescents during the COVID-19 pandemic (23, 24). Girls who previously had a low level of PA showed less of a tendency than boys to further decrease their level of PA, revealing a greater resilience in girls (25). It is plausible that boys who used to actively engage in organized outdoor team sports had many of their activities canceled due to the pandemic (26) and turned to more recreational screen time and sedentary behavior (27). Paradoxically, some studies of obesity have reported a higher prevalence of overweight and obesity in boys than in girls (28, 29), whereas others have demonstrated the opposite (30, 31). Furthermore, gender disparities have also been observed in weight perception and the motivation for weight management, which may influence the role of peer relationships in social networking. Compared with boys, girls have been reported to more frequently experience weight problems and have a stronger incentive to meet appearance-related goals (32), resulting in a higher proportion of same-gender network members in the promotion of PA (33). Jacob Miguel Vigil suggested that gender-specific contexts in relationship functioning and attraction preference may translate into differences in perceptions, initiation, engagement, and maintenance and, consequently, affect the process of behavior dissemination within a social network (34). Therefore, gender-related attributes and traits are particularly necessary when implementing social network interventions for adolescents. However, real-world networks often present a challenge in terms of directly understanding the mechanism of peer influence underlying such a gender context due to the great expense and effort involved in network data collection (35). It remains unclear how social networks may be exploited in a gender-specific manner to promote positive adolescent health behaviors.

Agent-based models (ABMs) have been used in social network interventions to explore promising approaches to addressing public health challenges (36, 37). These models are useful as they simulate dynamic patterns of adaptive behavior and identify the key criteria of influential agents (38). To understand the complex process of social interaction, ABMs allow agents to have a specific influence on others who connect with themselves while maintaining the attributes and characteristics of each agent in the production of population health disparities (i.e., gender differences). Moreover, as the social influence of environmental exposure on health can occur over time (39), ABMs provide a heterogeneous environment for simulating temporal variability, which enables vertical causality tests and long-term assessments of public health policies.

Objective of this study was to examine the effectiveness of centrality-based methods integrated with of gender contexts in a social network intervention to improve adolescent's health behavior. We developed an ABM to support network-based targeting methods and generate gender-specific health behavioral change. The social network structure used typical features with short average distances and a high degree of clustering to represent the small-world characteristics often found among adolescents (40). The network intervention focused on different selection criteria for the initial influential agents, as measured by four centrality indicators (i.e., betweenness centrality, closeness centrality, eigenvector centrality, and PageRank). To parameterize the model, we included adolescent health information, including gender, body mass index (BMI), and physical fitness (PF) test status, from both real-world datasets and other evidence from the literature for propagation details.

In regarding to the spread of health behavior, we sought to gain a greater understanding of how gender disparities in health performance and social interaction differentially determine the efficacy of school social network interventions. Specifically, we addressed the following research questions: Q1. what is the most effective centrality method in the general population? Q2. Do girls have a higher efficiency than boys? Q3. What is the most effective centrality method in girl's and boy's groups, respectively?

Following our conceptual framing and extant research, we hypothesized that the general spread of health behavior is expected to be faster under eigenvector-like centrality conditions than under betweenness or closeness centrality conditions (H1), with a higher efficiency in girls than boys (H2). In boys, an intervention based on the eigenvector centrality method is expected to be more effective than interventions based on other centrality-based methods (H3-a). Meanwhile, in girls, an intervention based on the page rank centrality method is expected to be more effective than interventions based on other centrality-based methods (H3-b).

## Methods

This section introduces the details of the social network simulation and analysis using our ABM. The demographic characteristics used to derive the parameter estimates are first illustrated intuitively, and then the social diffusion dynamics that are assumed for the propagation of health behavioral change in social networks are presented. We then discuss the construction and implementation of a synthetic network. Finally, we define the centrality measures used for the comparison of gender-specific effectiveness by networking.

### Population for Model Design

Data were collected in November 2018, November 2019 and November 2020, from three rounds of a national student physical fitness test for junior middle school students at one school in downtown Tianjin, China. Two hundred and thirty-four participants (113 girls, aged 13–14 years) completed both rounds of the test and had complete health-related information of height and weight, which were used to assess the BMI (i.e., underweight, normal weight, overweight, or obese) and physical fitness test status (i.e., excellent, good, fair, or fail). The BMI and physical fitness status were defined according to national student physical health standards for Chinese school-age children based on gender- and age-specific criteria (41). Participants were classified as overweight and obese according to the 85 and 95th percentiles of BMI, respectively. The results of multiple physical tests were used to determine student's physical fitness (e.g., 50-meter run, standing long jump, vital capacity, pull-ups for boys, and 1-min sit-ups for girls). Descriptive and correlational analyses were performed using SPSS software (IBM, Armonk, NY, USA). Z-tests were used to examine statistical significance of proportional differences. Chi-square tests were used to examine the difference in mean BMI change, overweight/obesity prevalence, and PF test performance by gender. All statistical tests were two-sided, and the statistical significance was set at a *P*-value < 0.05.

### Recognizing Gender Context in Behavior Patterns and Peer Network Formation

A higher prevalence of overweight/obesity in boys has been found consistently in previous studies of school-aged children in China (23, 28) and other Asian countries (42, 43). The reasons for this gender difference have been attributed to behavioral determinants, including diet (30, 44), PA (29, 45), weight perception (46, 47), motivation, and peer interaction (48), all of which may be key factors affecting the process of peer networking for health-related behavior promotion. Generally, boys are reported to consume more unhealthy food (e.g., high-energy fast food, sugar-sweetened food, and high-carbohydrate drinks) and show less preference for vegetables than girls. However, boys have more motivation and greater self-efficacy for PA improvement, especially in rigorous competitive PA and team sports, and are less likely than girls to be dissatisfied with their weight, which is a vital starting point to overcome perceived barriers to health behavioral change (49). Nevertheless, some studies have shown that girls place a higher value on adapting to social standards in the context of motivation for weight loss or physical appearance (32), indicating that adolescent girls may show greater adherence to school-based interventions.

Evidence shows that the effects of within-gender interactions are stronger than those of between-gender interactions (50, 51). A recent study of the heterogeneity of peer effects according to gender showed that girls are more sensitive to peer influence and are more influential than boys (52), implying that peer networks comprising only girls may be more likely to promote positive health behavior than those of boys. Moreover, girls may favor smaller group sizes (53, 54) for peer friendships and affiliations related to internalizing behaviors than do boys. However, an investigation of middle-aged adult PA reported no significant gender differences in the average network size (33).

Based on the distinctive social demands and relational constraints on behavioral patterns described above, we translated these gender features into probabilities for behavior in the ABM. Consequently, each gender was considered as an independent target in the follow-up intervention. This approach explicitly indicates disparate parameters of the network structure (i.e., gender rewiring proportion), the diffusion rate of health behavioral change (i.e., spread chance) and the perseverance likelihood of health behavior after the intervention (i.e., resistance chance) within our simulation model.

### Building the Agent-Based Model

An agent-based social network model was built using the Netlogo platform (55) to explore the efficiency of different network-based intervention strategies and determine which strategy is superior in terms of the functionality and flexibility of its implementation (35). For this model, a key assumption was that influential agents could spread their health behavior to their connected neighbors with a gender-specific probability. Thus, the number of influenced agents increased, and the positive effect spread through the social network. Meanwhile, all influenced agents had a gender-specific chance of persistence, which implied that once an influenced agent failed to persevere (relatively small chance), they would reenter the process of diffusion. Ultimately, our goal was to influence as many agents as possible, resulting in influenced agents maintaining a stable standard.

**Figure 1:**
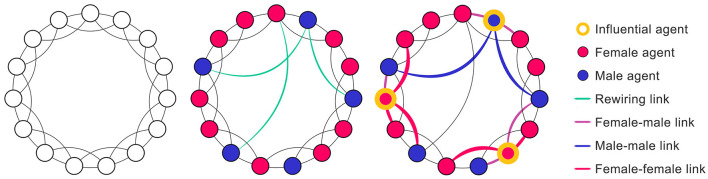
The building process of the Watts–Strogatz small-world model with gender contexts.

The social network was composed of a certain number of nodes (i.e., 234 agents equal to the number of participants) and links between them, with an appropriate structure indicating how these agents were connected. Different sets of initial influential agents contributed to a diverse range of propagation patterns based on the network structure. As the small-world property is often found in the social networks of adolescents (40), we relied on the well-known Watts and Strogatz (WS) (56) small-world model to generate the social network, assuming that small-world properties were always expressed in our simulation. These networks with short average distances between nodes (average path length, APL) and high clustering coefficient (CC) were created by a signature rewiring conduction (17), which maximized the optimal opportunities for influential nodes to propagate the desired influence among other nodes. The algorithm was first constructed by creating a ring of nodes, in which each node linked to two neighboring nodes on either side (four links in total). Then, each link was rewired with a certain probability. In our model, the principle of the rewiring process rewired the endpoint of a link with a small probability *p* (0.01 ≤ *p* ≤ 0.1) and considered the gender attributes of connected agents (Figure 1). The final proportion of links had a certain gender-specific probability (i.e., 41% for male-male, 38% for female-female, and 21% for male-female links), and the global social network satisfied the small-world properties (CC = 0.43, APL = 4.8). We used some parameters reported in previous studies to construct the network. For instance, according to the study of Rahmatollah et al. (57), the reciprocity rate was set at 0.54, with the degree of nodes set at ~12 and the clustering coefficient set at 0.42. In Framingham Heart's Study (58), the clustering coefficient was set at 0.66, and the reciprocity rate was 0.57 for males and 0.71 for females, respectively, based on data from the Add Health study (59).

Four centrality-based conditions were tested in the social network intervention (i.e., betweenness centrality, closeness centrality, eigenvector centrality, and PageRank), and the agents in the top 15% of centrality were assigned as influential agents initially, equal to the scale reported in other studies (27). Due to the uncertainty in selecting influential agents, we simulated the spread dynamics of each conditional scenario via hundreds of independently repeated experiments that used the same parameters and then averaged the outcomes to provide a single value for each condition.

Based on data from previous studies, the within-gender spread chance was higher than the between-gender spread chance, whereas the spread chance was relatively greater for females than males (i.e., 0.2 for male-male link, 0.25 for female-female link, and 0.1 for male-female link). Furthermore, regarding the perceived barriers and PA preference, the chance of resistance after being influenced was lower for males than for females (i.e., 0.1 for males, 0.2 for females). Therefore, in these experiments, we emphasized the general qualitative features of the results rather than the precise measures of diffusion rate to facilitate network intervention refinement and adolescent health improvements in future practice (40). An overview of the model parameters is presented in Table 1.

**Table 1:** Overview of parameters used for the social network ABM.

**Feature**	**Variable name**	**Value**
Small-world property	Clustering coefficient	0.43
	Average path length	4.81
Link proportion	Male-male	0.41
	Female-female	0.38
	Male-female	0.21
Influence agent	Initial influence proportion	0.15
Spread chance	Male-male	0.20
	Female-female	0.25
	Male-female	0.10
Resistance chance	Male	0.10
	Female	0.20

### Definitions for Centrality Measure

Certain types of centrality parameters traditionally have been considered in social network predictions (60). It is widely accepted that different applications require different centrality measure definitions (61). Thus, we briefly introduced these four centrality measures to further determine the effective centrality-based strategies for social network interventions.

Betweenness centrality is calculated as the number of the shortest paths between the members of each pair that pass through the current agent in sum (20). It depends on the extent to which an agent participates in information flow conducive to dissemination within the network intervention. In our experiment, a higher betweenness centrality agent implied a mediating role of social relationships between other indirectly linked agents, groups or subgroups. Once this agent is removed from a network, the diffusion of information may be disrupted, as the entire subgroup would be isolated from the intervention.

Closeness centrality is defined as the inverse of the average of the distances to all other agents (62). It indicates the reach of the desired influence of the diffusion speed and accessibility within a network-based intervention. It may be effective when selecting an agent with high closeness centrality as the initial influencing agent, as it disseminates the message through the fewest paths to reach all social network members.

Eigenvector centrality, devised by Phillip Bonacich (63), describes the amount of influence an agent has on a network. The importance of this influence depends on the number of linked agents (i.e., the degree of the node) and the value of the linked agents (i.e., the weight of the node), indicating that agents connected to other agents who are more well-connected will have a higher eigenvector centrality score. In our implementation, we distributed the initial influential values to each agent and designed rules to update the influential values iteratively until stable influential values were obtained (64). Eigenvector centrality was normalized so that a value of 1 was the highest eigenvector centrality an agent could take.

PageRank, a variant of eigenvector centrality, is a measure of the proportion of time that a message transferred randomly through the network spends at an agent (65). The message has an equal chance of taking any of the link edges and moves around the network completely randomly 15% of the time. The agent has a higher PageRank when connected to a small number of agents that are more important than themselves than when connected to a large number of agents that are less important than themselves. However, PageRank was defined for all networks, regardless of connectivity, which distinguished it from eigenvector centrality. We treated all links as undirected links in our simulation, and the sum of all PageRank values should be ~1.

## Results

In this section, statistical analyses of the empirical data are first presented. We added an additional condition to validate our model by randomly selecting influential agents. In the main analyses, we created a centrality-based factorial design to investigate the contribution of various network features to the change in the average diffusion speed of a synthetic population.

### Preliminary Analyses

The results of the statistical analyses indicated a marked change in the prevalence of overweight and obesity in Chinese adolescents due to COVID-19-related school closures. As shown in Table 2, there were significant increases among all participants in both the mean BMI (pre vs. post-lockdown: +0.52 vs. +1.39 kg/m^2^, *p* < 0.001), and the overall overweight/obesity prevalence (pre vs. post-lockdown: +0.43 vs. +5.13%, *p* < 0.001). It is worth noting that gender differences included a greater increase in BMI in boys than in girls (+1.48 vs. +1.29 kg/m^2^, *p* = 0.036), and a higher total prevalence of overweight/obesity in boys than in girls (47.11 vs. 40.71%, *p* = 0.047). Approximately 55.34% of boys were overweight or obese, indicating higher odds of an unhealthy BMI status, which may be associated with the difference in hormone biology as well as certain social and cultural factors (66). However, the increase in the prevalence of overweight/obesity was markedly higher in girls (pre vs. post-lockdown: +0.00 vs. +8.85%, *p* < 0.001) than in boys (pre vs. post-lockdown: +0.83 vs. +1.65%, *p* < 0.001). In addition, correlation analysis demonstrated that BMI and physical fitness scores were negatively correlated (-0.589, *p* < 0.05), which may partly explain the unsatisfactory outcomes of the increased number of physical fitness test failures. In conclusion, the many adverse effects of COVID-19-related lockdowns on BMI and obesity in adolescents confirmed the urgent need for health behavior promotion strategies in school settings aimed at increasing adolescent's fitness to pre-pandemic levels.

**Table 2:** Descriptive and correlational analyses of the study population.

		**Total**	**Girls**	**Boys**	***P*-value^a^**
Age		13.35	13.35	13.36	0.878
Number (*n*, %)		234	113 (48.29%)	121 (51.71%)	0.274
Mean BMI (kg/m^2^)	2018.11	21.42	20.62	22.17	0.012
	2019.11	21.94	21.30	22.54	0.048
	2020.11	23.32	22.59	24.01	0.036
Overweight/obesity prevalence (%)	2018.11	38.46	31.86	44.62	0.023
	2019.11	38.89	31.86	45.45	0.005
	2020.11	44.02	40.71	47.11	0.047
Unhealthy weight in PF test failure (%)	2018.11	66.67	32.60	67.40	0.001
	2019.11	75.00	40.00	84.21	0.929
	2020.11	81.25	50.00	88.46	0.008
BMI and PF test failure Pearson Correlation	2018.11	−0.494^***^	–	–	<0.05
	2019.11	−0.540^***^	–	–	<0.05
	2020.11	−0.589^***^	–	–	<0.05

a *Difference between boys and girls*;

*** *Significant at P-value < 0.05*.

### Model Validation

For model validation, an extra experiment was performed in which the initial influential agents were selected at random using the same model parameters and compared with the centrality-based network methods. We focused more on the diffusion speeds of various targeting interventions at the population level than the success rate of each condition. The statistical analysis revealed (Table 3) that, on average, the diffusion speed was lower under the random condition (5.22 % per tick, *p* < 0.05) than under the centrality-based conditions (6.17% per tick, *p* < 0.05), and there were no significant differences in the average final success rate between the random condition (98.72%, *p* < 0.05) and the centrality-based conditions (98.89%, *p* < 0.05). These findings indicate the credibility of the simulated centrality-based interventions based on the ABM approach.

**Table 3:** Descriptive statistics for the social network interventions.

**Variables**	**Random**	**Betweenness**	**Closeness**	**Eigenvector**	**PageRank**
Mean	–	0.04	0.21	0.33	0.43
Maximum	–	0.15	0.25	1.00	0.70
Minimum	–	0.00	0.17	0.07	0.01
Success rate (%)	98.91	98.92	98.71	98.96	98.97
Diffusion speed (% per tick)	5.22	6.16	5.98	6.16	6.37
Female (% per tick)	2.85	3.85	3.57	3.54	3.76
Male (% per tick)	2.81	2.96	2.86	2.92	3.21

### Model Analyses

**Figure 2:**
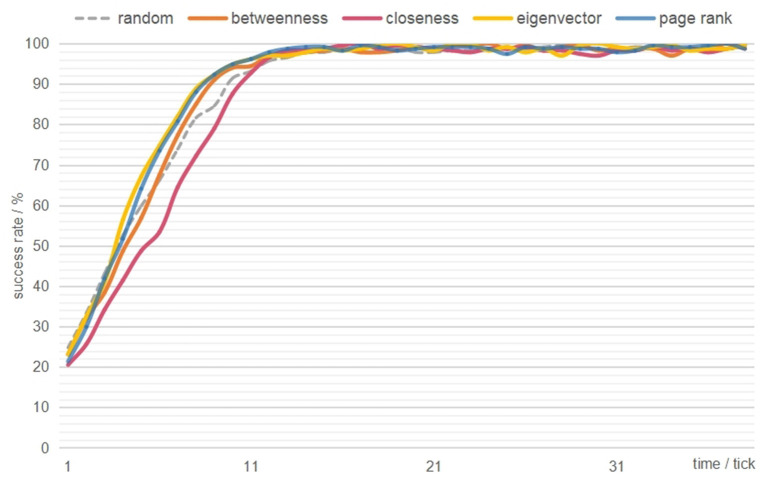
Intervention outcomes of the random and four centrality-based conditions.

To test the effectiveness of a hypothetical social network intervention for all adolescents (H1), the four centrality-based simulated conditions (i.e., betweenness centrality, closeness centrality, eigenvector centrality, PageRank) were compared (Figure 2). The average diffusion speed of the PageRank condition (6.37% per tick, *p* < 0.05) was higher than the average diffusion speed of the other targeting conditions, which indicates that an agent who has high prestige among their peers might more efficiently push the entire community toward a change.

**Figure 3:**
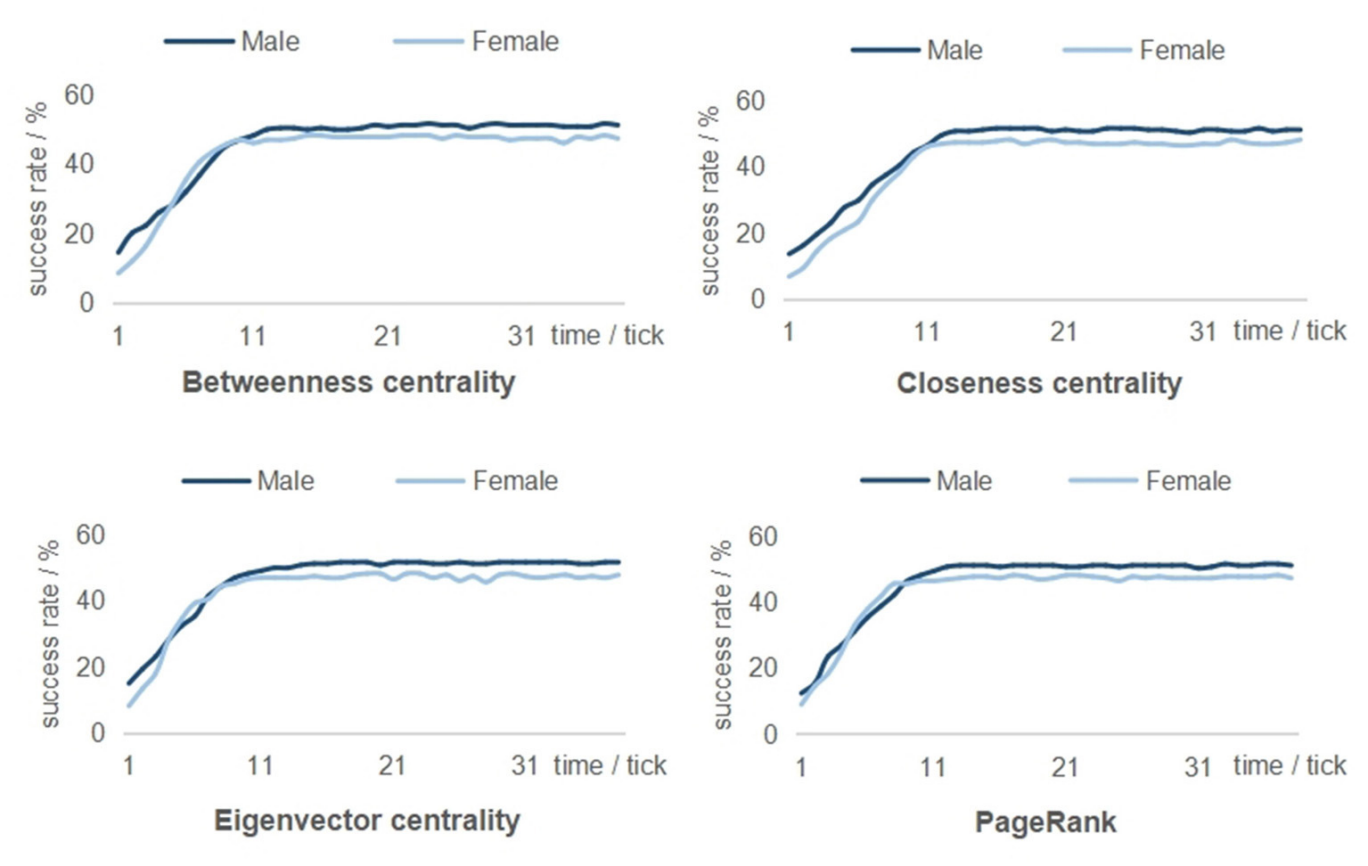
Gender differences under four centrality-based interventions.

To answer the research question on gender performance (H2), the simulation result of each centrality was recorded (Figure 3). In contrast to our expectation, the diffusion speed under the random condition showed almost no difference between the genders (male 2.81% vs. female 2.85% per tick, *p* < 0.05). In contrast, based on the centrality-based targeting methods, females always exhibited an advantage over males in the average diffusion speed (male 2.99% vs. female 3.68% per tick, *p* < 0.05). This finding indicates that influential agents selected at random may unconsciously reduce the gender disparities in behavioral change.

To determine a gender-specific network-based strategy (H3-a/b), betweenness centrality was assumed to be the most effective targeting method for girls, with the highest diffusion speed (3.85% per tick, *p* < 0.05), followed by the PageRank method (3.76% per tick, *p* < 0.05). The closeness centrality (3.57% per tick, *p* < 0.05) and eigenvector centrality (3.54% per tick, *p* < 0.05) methods provided a relatively slight advantage for adolescent girls. However, the simulated results by the same targeting strategies showed no coherence in boys. PageRank (3.21% per tick, *p* < 0.05) outperformed other conditions, followed closely by betweenness centrality (2.96% per tick, *p* < 0.05) and eigenvector centrality (2.92% per tick, *p* < 0.05). Closeness centrality method (2.86% per tick, *p* < 0.05) still failed to show potential to propagate health-related behaviors among adolescent boys.

## Discussion

This paper provides evidence for the efficiency of centrality-based strategies for adolescent health behavior promotion. To the best of our knowledge, this is the first study to explore a social network ABM that supports the expression of a small-world property and gender-specific attributes. Based on the social network theory, the health behavior modes of adolescents are often determined by massive connections and central positions within the network (67). However, the current practice of network-based interventions does not recognize the gender disparities in behavioral patterns and emotional preferences, exposing girls still lag behind in certain types of health-related performance, such as PA engagement. Therefore, identifying influential spreaders may help enhance the diffusion of positive health behavior in future school social network interventions.

By focusing on the diffusion speeds of different selection criteria for initial influential agents, this study brings insights into two aspects. First, we found that PageRank was the best method among all centrality conditions at the population level (H1). PageRank is a mathematical algorithm applied by search engines use to determine the importance of a website. It can also be more practically extended to determine social influence (68) and analyze citation networks (69) and other issues (70). Contrastively, the four centrality measures we used can be grouped into two categories of social network prediction. Betweenness and closeness centrality both consider influential agents and depend on their capabilities of controlling information flow in the global network, whereas the eigenvector-like centralities (i.e., eigenvector centrality and PageRank) calculate the importance of an agent based on both the quantity and quality of its neighbors. With a distinctive emphasis on the network structure, these centralities may be regarded as generalizations of the centralities of static networks (71), with potential uses in a wide range of applications. For instance, it has been proposed that betweenness centrality is appropriate to prevent the spread of negative health behavior in a network-based intervention (72). However, the promotion of positive health behavior based on the closeness centrality method might be an efficient strategy (67), as the energetic message or opinion (e.g., PA) would reach the entire network easily without any subgroups being excluded from the intervention (73). Significantly, eigenvector-like centralities that take immediate, mediative, and global effects of social interactions into account (74) have been shown to be successful and effective at assigning centrality weights to the nodes in a network to determine the influence of social peers (71).

Meanwhile, the advantage of closeness centrality was not shown in our simulation, which is similar to the effect reported by Van Woudenberg et al. (75). One explanation for this finding is that closeness centrality agents that closely connect to all members of a network often represent the geometric center of the network. It is possible that such influential agents are not effective in “persuading” when bridging and bonding in a community that is separated into gender subgroups. Children prefer to associate with “same-gender” peers beginning at an early age (76), and this continues through adolescence. A previous study found that adolescents aged 12 to 16 were approximately three times more likely to have “same-gender” friends than “other-gender” friends (77). Building on this conception, the gender-specific subgroups in an adolescent network are more likely to be affected by their central leaders, such as the PageRank agents, rather than closeness centrality agents that lack a high-status despite being close to the entire network. This finding is also consistent with the performance disparity between the closeness and betweenness centrality conditions, as betweenness centrality emphasizes the high level of connectivity of influential agents to multiple subgroups.

Our second finding demonstrated the necessity for gender-specific strategies with different behavioral features and related performance in school social network interventions (H3-a/b). We observed that the PageRank centrality method was more efficient in males, while the betweenness centrality method was superior for network-based interventions in females. Studies have reported gender differences in behavioral patterns such that girls withdraw from boy's more aggressive style of PA engagement (78), which may explain the same-gender preference among girls. However, health promotion programs in most schools have not recognized the gender-related barriers to and opportunities for PA-related behaviors, including a lack of diverse resources and activity categories. Furthermore, emotional preferences enforce the distinctive mechanism of peer influence, as adolescent girls have a stricter definition of health, especially weight status. Those with a higher weight often experience more pressure to be slim and have been identified as a unique cluster, based on homophily and contagion assumptions, in social network studies of obesity (79). Therefore, our simulation result supported that the betweenness centrality agents may play a vital mediating role between different subgroups. This finding creates a positive intention for health-related behaviors associated with weight loss in adolescent girls, especially for those with an unhealthy weight. The betweenness cetrality method may drive subsequent behavioral change when female subgroups remain stable.

In contrast, boys are viewed as stronger and more apt than girls to participate in MVPA, such as cycling and running. It has also been argued that PA may be an additional leisure-time requirement for wellbeing in the daily lives of boys, making them more likely to continue PA behavior after achieving a weight-loss goal (80). A recent survey showed that more boys than girls reported unchanged levels of PA during COVID-19-related lockdowns, which resulted in more stable PA behavior in boys (81). These findings may be due to the originally autotelic purposes for participating in sports and PA-related behaviors among many adolescents. For instance, boys have been shown to participate in sports mainly for competition, whereas girls participate mainly for the social benefits. To some extent, the unique appeal of competitive MVPA to boys gives novel insights into network-based intervention strategies. Therefore, to promote positive health behavior to boys, we recommend that influential agents within male subgroups may be more effective than popular agents outside the subgroups at reaching the overall network with health-related messages, thus highlighting the reliability of eigenvector-like centrality agents in social network interventions. Notably, the idea that the selection criteria of eigenvector-like centralities provide an effective response to the social network intervention at the population level does not necessarily overemphasize that being active with friends is socially desirable (82).

### Strengths and Limitations

The present study has several strengths. At the theoretical level, we have provided cross-sectional evidence for the negative effects of school closures and quarantine time on adolescent health outcomes during the COVID-19 pandemic, including changes in BMI and overweight/obesity prevalence and the association between higher BMI and worse physical test performance among Chinese junior middle school students. Second, based on the literature we reviewed, this is the first study to explore gender-specific recommendations for implementing a social network intervention to address adolescent health behavior promotion in the school environment. We considered gender contexts in terms of the effects of multiple behavioral patterns and emotional preference on PA and weight loss using an ABM. Our investigation explicitly shed light on the roles of different strategies targeting influential agents through the comparison of four centrality-based network measures.

Nevertheless, this study has several limitations. First, there are data shortcomings that limit the applicability of our findings. The health-related data we used to capture a generally negative health impact due to COVID-19-related lockdowns was based on the overall perceptions of a variety of factors, such as family support and the physical environment. However, these data did not specify social cues, such as behavior links and peer interactions. Consequently, we used the WS small-world model to generate a synthetic network with parameters that were mainly based on data from the literature. Future studies assessing social network features in the real world, and the peer selection cues upon which they are based, should seek to unravel these assumed mechanisms. Similarly, we did not assess the extent to which the diffusion process and persistence chance differed according to gender, as these contexts would lead to inescapable effects on the simulation. More empirical data on detailed aspects of gender-specific health behaviors related to social networking are needed to further understand whether the behavior preference due to gender disparities results in the formation of group-related health behaviors, and more specifically, what types of PA-related behavior are preferred in these group settings.

Secondly, based on the mathematical characteristics of the model, the outcome of the ABM showed an initial increase and then reached a state of equilibrium after a certain amount of time in the simulations. Caution may be warranted when interpreting the absolute increase in positive health behavior development in reality. For instance, studies have reported a positive association between health behavior (i.e., PA promotion) and the built environment of the school, which indicates that certain physical factors, such as physical education facilities, playground characteristics, and the presence of green spaces, may affect the adoption of healthier PA behaviors by students (83). In addition, we assessed peer influence from the perspective of network structure, although this structure may also be based on many other social factors (e.g., parents, teacher relationships, and sports interests). Therefore, more extensions are possible to improve the model. For example, the contribution of physical and social environment features may be combined to account for gender-specific contexts, and multi-environmental influence modeling may be explored for adolescent health behavior promotion.

## Conclusions

The COVID-19 pandemic has had an adverse effect on adolescent health globally and has emphasized the urgent need for health behavior promotion when resuming school attendance. Over the last few years, social network interventions have been successfully applied to address adolescent health issues such as obesity and physical inactivity (84, 85) through social influence. This study firstly demonstrated a significant gender difference, in that girls showed a greater increase in the prevalence of overweight and obesity compared with boys. To extend our understanding of social interactions in complex network systems, we confirmed that incorporating gender-related contexts in a social network ABM is a promising approach to disseminating the positive health behavior information. The results suggested that the PageRank centrality method had the highest efficiency in the general and male-only simulation groups, while in female-only groups, betweenness centrality offered potential advantages over the other centralities. As social media has especially become popular in the adolescent age groups nowadays, we reinforced the need to encourage the social influencers and to support peer connectedness via not only real-life but also mobile social network, which might mitigate some of the negative effects of physical distancing (86). These findings indicate that schools and public health organizations should maintain heightened sensitivity to social stimuli, and develop gender-specific strategies and policies to improve adolescent's health behavior in the post-pandemic era.

## Data Availability Statement

The raw data supporting the conclusions of this article will be made available by the authors, without undue reservation.

## Ethics Statement

The studies involving human participants were reviewed and approved by the Medical Research Ethics Committee of Tianjin University (TJUE-2021-174). Written informed consent to participate in this study was provided by the participants' legal guardian/next of kin.

## Author Contributions

SZ: conceptualization, data curation, methodology, validation, formal analysis, visualization, writing of original draft, and review and editing. TX: methodology, software, visualization, and manuscript review and editing. JH: project administration, resources, supervision, and manuscript review and editing. All authors contributed to the article and approved the submitted version.

## Funding

This research is supported by the Stable Support Programme for Higher Education by Shenzhen R&D Funds (Grant Nos. GXWD20201230155427003-20200803172955008).

## Conflict of Interest

The authors declare that the research was conducted in the absence of any commercial or financial relationships that could be construed as a potential conflict of interest.

## Publisher's Note

All claims expressed in this article are solely those of the authors and do not necessarily represent those of their affiliated organizations, or those of the publisher, the editors and the reviewers. Any product that may be evaluated in this article, or claim that may be made by its manufacturer, is not guaranteed or endorsed by the publisher.
